# Prevalence of chronic kidney disease and associated factors among adult diabetic patients: a hospital-based cross-sectional study

**DOI:** 10.3389/fepid.2024.1467911

**Published:** 2024-11-19

**Authors:** Kibrom Aregawi, Getachew Kabew Mekonnen, Rebuma Belete, Winner Kucha

**Affiliations:** ^1^ Department of Medical Laboratory Sciences, College of Medicine and Health Science, Adigrat University, Adigrat, Ethiopia; ^2^ School of Medical Laboratory Sciences, College of Health and Medical Sciences, Haramaya University, Harar, Ethiopia

**Keywords:** prevalence, chronic kidney disease, glomerular filtration rate, diabetes mellitus, Ethiopia

## Abstract

**Background:**

Chronic kidney disease (CKD) has a significant impact on public health with a high morbidity and death rate. Most diabetic patients, in the course of their lives, develop diabetic kidney disease. In the least developed nations, its size is outstripping itself. This study aimed to determine the prevalence of chronic kidney disease and associated factors among adult diabetic patients.

**Methods:**

A hospital-based cross-sectional study was conducted on 328 adult diabetic patients from 1 December 2023 to 4 April 2024 at the Ayder Comprehensive Specialized Hospital, northern Ethiopia. A systematic random sampling method was utilized to select the study participants. Pretested structured questionnaires were used to collect sociodemographic, economic, and behavioral/lifestyle factors. Medical records were also reviewed to collect clinical data. Creatinine analysis was performed by kinetic alkaline picrate method and Chronic Kidney Disease Epidemiology Collaboration 2021 equation was used to calculate the glomerular filtration rate from the serum creatinine, age, and sex. Proteinuria was determined by using the dipstick semiquantitative method. Data were entered and analyzed using SPSS version 29. A variable with a *p*-value of <0.25 in bivariate logistic regression analyses was analyzed in multivariate logistic regression to identify the associated factors. In multivariable logistic regression, a variable was deemed statistically significant if it had a *p*-value <0.05. Associations were presented as odds ratio (OR) along with 95% confidence intervals (CIs).

**Results:**

The prevalence of chronic kidney diseases in adult diabetic patients was 26.5% (95% CI, 21.8%–31.7%). About 5.2%, 12.5%, 7.3%, 0.9%, and 0.6% had stage 1–5 chronic kidney diseases, respectively. Hypertension [adjusted OR (AOR) = 2.390; 95% CI, 1.394–4.099, *p* = 0.002], >10-year duration of diabetes (AOR = 2.585; 95% CI, 2.321–5.807; *p* = 0.001), and family history of kidney diseases (AOR = 2.884; 95% CI, 1.338–6.218; *p* = 0.007) were associated factors of chronic kidney diseases.

**Conclusions:**

The study revealed that one in four diabetic patients had chronic kidney disease. Special attention should be given to patients with family history of CKD, long duration on diabetes, and concomitant hypertension.

## Introduction

Chronic kidney disease (CKD) is a condition where there are abnormal alterations in the functioning or structure of the kidneys. This is indicated by a glomerular filtration rate (GFR) that is below 60 ml/min/1.73 m^2^ or the presence of proteinuria, or both, for a duration of more than 3 months (1). According to the guidelines of the Kidney Disease Improving Global Outcomes, CKD is classified into five stages. Individuals with an estimated GFR (eGFR) of ≥90 ml/min/1.73 m^2^ are grouped into stage 1 with a normal kidney function. The second stage is when eGFR is 60–89 ml/min/1.73 m^2^ with a mild decrease in kidney function. The third stage is separated into two categories: stage 3a (eGFR 45–59 ml/min/1.73 m^2^) and stage 3b (eGFR 30–44 ml/min/1.73 m^2^). The fourth stage of CKD occurs when the eGFR drops to 15–29 ml/min/1.73 m^2^, and the final stage occurs when the GFR falls below 15 ml/min/1.73 m^2^ (2).

Most diabetic patients will eventually develop diabetic kidney disease (DKD) (3). The pathophysiology of DKD is multifactorial and involves several key mechanisms. Hyperglycemia is the primary cause of DKD. High blood sugar levels directly damage the kidneys’ filtering system over time. Altered tubuloglomerular feedback, renal hypoxia, and activation of the renin–angiotensin system contribute to the hemodynamic changes. Hyperglycemia also triggers inflammation, with increased levels of cytokines, chemokines, oxidative stress, and advanced glycation end-products also playing a role in DKD pathogenesis (4–7).

Diabetes and elevated blood pressure are the most frequent causes of CKD in most adults. Heart disease, a family history of CKD, having inherited renal problems, past kidney injury, being older, and being obese are additional risk factors for CKD (8).

Chronic kidney disease affects more than 10% of the world's population. The disease was ranked 16th among the major causes of death in 2016, and it is anticipated to rise to 5th by 2040 (9). It is estimated that 850 million people worldwide suffer from kidney disease, with the majority residing in lower middle income and low income countries. Globally, CKD resulted in approximately 3.16 million deaths and 76.5 million disability-adjusted life years (10).

In Ethiopia, CKD is also a major public health problem (11) with prevalence ranges from 9.3% to 25.9% in diabetes mellitus (DM) patients (12). Most of the studies on CKD were modification of diet in renal disease (MDRD) and Cockcroft–Gault equations to estimate GFR (13–15). However, the Chronic Kidney Disease Epidemiology Collaboration (CKD-EPI) equation is known to have the highest accuracy in estimating GFR and is currently recommended for use to estimate GFR by Kidney Disease: Improving Global Outcomes (KDIGO) clinical practice guidelines (2). Thus, this study was conducted to determine the prevalence of CKD using the CKD-EPI equation and to identify associated factors among diabetic patients.

## Methods and materials

### Study design, area, and period

A hospital-based cross-sectional study was conducted at Ayder Comprehensive Specialized Hospital from 1 December 2023 to 4 April 2024. The hospital is located in Mekelle, in the Tigray Region, 783 km north of Addis Ababa, Ethiopia's capital. The hospital has more than 500 inpatient beds with 36 Intensive care units and more than 3,600 employees in 2022. It offers a wide range of medical treatments, such as dialysis, cancer treatment, and neonatal care, to both inpatients and outpatients of all ages. In addition, it acts as a research and teaching hospital for medicine and other health sciences. During the time of data collection, there were about 2,300 diabetic patients receiving care at the hospital's diabetic clinic.

### Study population

The study included adult patients (≥18 years) who had been previously diagnosed with diabetes mellitus and attended the hospital's follow-up diabetes clinic during the study period. Patients with diabetes who were pregnant, critically ill, had incomplete clinical and laboratory data, or had a short follow-up period (<6 months) were not included in the study.

### Sample size determination

A minimum sample size of 328 was determined using a single population proportion formula, *n* = 1/*d*^2^ × [(*Z_α_*_/2_)^2^ × *P* (1 − *P*)], where *n* is the sample size, *Z* = 1.96 [95% confidence interval (CI)], *P* = 0.263, the proportion of chronic kidney disease among DM patients from a previous study (15), and *d* is the assumed margin of error (5%), and a 10% non-response rate was considering. Sample size was also calculated for selected variables and found to be below 328.

### Sampling procedure

According to the hospital report, 2,300 diabetic patients were in regular follow-up at the diabetic clinic. By taking their medical registration book as a sampling frame, interval size (*k*) was calculated by dividing the total number of diabetic patients on a follow-up by the desired sample size (i.e., *k* = 2,300/328 = 7). By using a systematic random sampling method, the participants were selected every 7 intervals from the list of medical registration book. If the *k*th or selected patients were not willing to participate in the study or did not fulfill the inclusion criteria, the next diabetic patient was recruited to the study.

### Data collection and laboratory measurements

Data on sociodemographic, economic, and behavioral/lifestyle factors were collected using pretested, structured questionnaires. Medical records of the patients were also reviewed for clinical history including types of DM, blood glucose levels, and a history of hypertension. The average blood pressure and blood glucose over the previous 3 months were utilized to calculate the participants’ blood pressure and glycemic status.

Blood pressure measurements were carried out using a manual sphygmomanometer after patients had been comfortably resting for at least 5 min. About 5 ml of venous blood specimens were drawn and processed, and the serum was separated for biochemical analysis. Serum creatinine analysis was carried out using kinetic alkaline picrate method on the Cobas C311 clinical chemistry analyzer. Participants with abnormal creatinine measurements were checked again for persistence after 3 months.

Fresh 10 ml urine specimen was collected from each participant to detect proteinuria using dipsticks. Participants with positive proteinuria were reexamined for persistence after 3 months.

The eGFR was calculated by using the CKD-EPI 2021 equation (16):

$$\mathrm{eGFR}=142\times \min(\mathrm{Scr}/k,1{)}^{\alpha }\times \max(\mathrm{Scr}/k,1{)}^{-1.200}\times 0.9938^{\mathrm{age}}\times 1.012(\mathrm{if}\;\mathrm{female})$$

where GFR is the glomerular filtration rate (ml/min/1.73 m^2^), min is the minimum of Scr/*k* or 1, max is the maximum of Scr/*k* or 1, Scr is the serum creatinine level (in mg/dl), *k* = 0.7 (females) or 0.9 (males), and *α* = −0.241 (females) and −0.302 (males).

### Operational definitions

• **Chronic kidney disease**: having eGFR of less than 60 ml/min/1.73 m^2^ and/or proteinuria, for at least 3 months (1).
• **Stages of CKD** (1):
  ○ Stage 1: persistent proteinuria with eGFR ≥90 ml/min/1.73 m^2^
  ○ Stage 2: persistent proteinuria with eGFR of 60–89.9 ml/min/1.73 m^2^
  ○ Stage 3a: eGFR 45–59.9 ml/min/1.73 m^2^ with or without proteinuria
  ○ Stage 3b: eGFR 30–44.9 ml/min/1.73 m^2^ with or without proteinuria
  ○ Stage 4: eGFR 15–29.9 ml/min/1.73 m^2^ with or without proteinuria
  ○ Stage 5: eGFR <15 ml/min/1.73 m^2^ with or without proteinuria
• **Proteinuria**: Presence of protein in urine indicated by manual urine dipstick result of 1+ or above (approximately greater than 30 mg/dl).
• **Regular physical activity**: Any kind of movement performed in a regular way to make the body refresh and active. This includes walking, running, swimming, cycling, wheeling, and others. It was assessed by asking the participants, “how often do you engage in physical activity regularly per week?”
• **Smoking habit**: If the respondents had an act of inhaling and exhaling the fumes of burning plants or other chemicals. It was assessed by asking the participants, “did you smoke a cigarette or other chemicals currently?” and “did you smoke before?”
• **Alcohol consumption**: Act of drinking alcoholic fluids. Assessed by asking the participants, “did you consume alcohol currently?” and “did you consume alcohol before?”
• **Alcohol ex-consumer**: A participant who did not consume alcohol in the last 1 year but previously did that.

### Data quality assurance

Data collectors and a supervisor received 1-day training on data collection instruments and study objectives to guarantee that the data quality was not compromised. Questionnaire was pretested using 5% of the total participants (17 patients) at the Mekelle General Hospital before data collection, and appropriate modifications were made. Close follow-up and supervision were carried out during the data collection period jointly by the first author and the supervisor. The collected data were reviewed and checked for its completeness before data entry. For creatinine analysis, the reaction was monitored every working shift by using both normal and pathological control samples. Quality control of the urine dipstick testing was also done in accordance with the standard operating procedure.

### Statistical analysis

The data were entered into SPSS version 29 for analysis. A descriptive statistic was used to describe the study participants and relevant variables. The associations between chronic kidney disease and all above mentioned variables were evaluated in bivariable logistic regressions. Those variables that showed a *p*-value of 0.25 or smaller in these bivariable regressions were further included in a multivariable logistic regression model. The final multivariable model only included variables that were significantly associated based on *p*-values ≤0.05. Adjusted odds ratios (AORs) and 95% CIs are reported to measure the strength of the associations. Hosmer and Lemeshow goodness of fit was typically utilized to assess model fitness.

## Results

### Sociodemographic characteristics

A total of 328 adult diabetic patients participated in the study. Of them, 169 (51.5%) were in the age group of ≤45 years old, more than half [191 (58.2%)] were female, 243 (74.1%) were urban dwellers, 289 (88.1%) were ethnic Tigrayans, 241 (73.5%) were married, 125 (38.1%) had primary education, and 205 (62.5%) had less than 3,000 Ethiopian birr in monthly income (Table 1).

**Table 1:** Sociodemographic characteristics of diabetic patients at the Ayder Comprehensive Specialized Hospital, northern Ethiopia, 2024 (*n* = 328).

Variables	Category	Frequency (%)
Age category (years)	≤45	169 (51.5)
	>45	159 (48.5)
Gender	Male	137 (41.8)
	Female	191 (58.2)
Residence	Urban	243 (74.4)
	Rural	85 (25.6)
Marital status	Married	241 (73.5)
	Single	84 (25.6)
	Widowed	2 (0.6)
	Divorced	1 (0.3)
Educational status	No formal education	106 (32.3)
	Primary school (1–8)	125 (38.1)
	Secondary school (9–12)	62 (18.9)
	College/University	35 (10.7)
Monthly income (ETB)	<1,000	205 (62.5)
	1,000–3,000	76 (23.2)
	3,001–5,000	35 (10.7)
	>5,000	12 (3.7)

### Clinical and behavioral characteristics

The proportion of patients with type 2 and type 1 diabetes mellitus was 181 (55.2%) and 147 (44.8%), respectively. Among the study participants, 97 (29.6%) had hypertension. The majority of the participants [200 (61%)] were classified as having normal BMI and 35 (10.7%) had family history of kidney disease.

Furthermore, 89 (27.1%) had a long duration of diabetes (>10 years), almost all [325 (99.1%)] never smoked, and 308 (93.9%) had no alcohol consumption habit. The majority [314 (95.7%)] had never engaged in regular physical exercise. About half of the participants 165 (50.3%) had normal systolic blood pressure (<120 mmHg), and the majority [239 (72.9%)] had normal diastolic blood pressure (60–79 mmHg) (Table 2).

**Table 2:** Clinical and behavioral characteristics of diabetic patients attending at the Ayder Comprehensive Specialized Hospital, northern Ethiopia, 2024 (*N* = 328).

Variables	Category	*N* (%)
BMI (kg/m^2^)	Underweight (<18.5)	28 (8.5)
	Normal weight (18.5–24.9)	200 (61.0)
	Overweight (25–29.9)	69 (21.0)
	Obese (≥30)	31 (9.5)
Type of diabetes	Type 1 diabetes mellitus	147 (44.8)
	Type 2 diabetes mellitus	181 (55.2)
Duration of diabetes (years)	<5	131 (39.9)
	5–10	108 (32.9)
	>10	89 (27.1)
Hypertension	Present	97 (29.6)
	Absent	231 (70.4)
Smoking habit	Smoker	3 (0.9)
	Non-smoker	325 (99.1)
Alcohol consumption	Alcohol consumer	13 (4.0)
	Alcohol non-consumer	308 (93.9)
	Alcohol ex-consumer	7 (2.1)
Regular physical activity (days/week)	None	314 (95.7)
	1–2	7 (2.1)
	3–4	6 (1.8)
	>5	1 (0.3)
Family history of kidney disease	Present	35 (10.7)
	Absent	293 (89.3)
SBP (mm Hg)	Normal (<120)	165 (50.3)
	Elevated (120–129)	50 (15.2)
	Stage 1 (130–139)	45 (13.7)
	Stage 2 (≥140)	68 (20.7)
DBP (mm Hg)	Normal (60–79)	239 (72.9)
	Stage 1 (80–89)	60 (18.3)
	Stage 2 (90–109)	29 (8.8)
FBG (mg/dl)	<150	146 (44.5)
	≥150	182 (55.5)
Proteinuria	Negative	259 (79.0)
	Positive	69 (21.0)

BMI, body mass index; DBP, diastolic blood pressure; FBG, fasting blood glucose; SBP, systolic blood pressure.

### Prevalence and stages of chronic kidney diseases

The overall prevalence of CKD was 26.5% (95% CI: 21.8%–31.7%), of which, 29 (8.8%) had eGFR <60 ml/min/1.73 m^2^ and 69 (21%) had proteinuria. From the total patients with CKD (87), 17 (5.2%), 41 (12.5%), 24 (7.3%), 3 (0.9%), and 2 (0.6%) were categorized as stage 1–5, respectively.

From the total patients with CKD (87), 11 (13%) had both impaired GFR (<60 ml/min/1.73 m^2^) and proteinuria, 13 (14%) had only impaired GFR (eGFR < 60 ml/min/1.73 m^2^), and 63 (74%) had only proteinuria.

### Factors associated with chronic kidney diseases among study participants

When it was analyzed with multivariate logistic regression, hypertension, >10 years duration of diabetes, and family history of kidney disease were independently associated factors for chronic kidney disease (*p* < 0.05).

Patients with concomitant hypertension were 2.390 (AOR = 2.390; 95% CI, 1.394–4.099; *p* = 0.002) times more likely to develop CKD compared with non-hypertensive patients. The odds of CKD was 2.585 (AOR = 2.585; 95% CI, 2.321–5.807; *p* = 0.001) times higher among patients who have had a longer (>10 years) duration of diabetes compared with patients having a short duration. Patients with a family history of kidney diseases were 2.884 (AOR = 2.884; 95% CI, 1.338–6.218; *p* = 0.007) times more likely to have CKD than having no family history of kidney diseases (Table 3).

**Table 3:** Bivariate and multivariate logistic regression analysis of factors associated with chronic kidney disease among adult diabetic mellitus patients at the Ayder Comprehensive Specialized Hospital, northern Ethiopia, 2024 (*n* = 328).

Variables	Category	Total number	CKD (%)		COR (95% CI)	AOR (95% CI)
			Yes (%)	No (%)		
Age (years)	≤45	169	12.8	65.2	1	1
	>45	159	13.7	8.3	0.720 (0.419–1.237)*	1.293 (0.598–2.794)
Gender	Male	137	12.2	55.0	1	
	Female	191	14.3	18.5	0.810 (0.518–1.338)	
Residence	Urban	243	19.7	47.6	1	
	Rural	85	6.8	25.9	0.923 (0.619–1.521)	
Ethnicity	Tigrinya	289	23.8	63.0	1	
	Afar	16	2.1	4.5	0.872 (0.697–1.011)	
	Amhara	9	0.3	2.4	0.975 (0.817–1.168)	
	Eritrean	12	0.3	3.0	0.678 (0.307–0.918)	
	Others	2	0.0	0.6	0.145 (0.017–0.308)	
Marital status	Single	84	12.3	34.0	1	
	Married	241	14.2	18	1.659 (0.877–3.118)	
	Divorced	1	0.0	11.5	1.677 (0.977–3.238)	
	Widowed	2	0.0	10.0	1.757 (0.975–3.148)	
Educational status	No formal education	106	8.2	24.4	1	1
	Primary (1–8)	125	12.8	31.0	1.357 (0.995–2.108)*	1.417 (0.972–2.066)
	Secondary (9–12)	62	3.4	10.3	1.559 (1.175–1.942)*	1.619 (1.174–2.206)
	College/University	35	2.1	7.8	1.757 (0.975–3.148)*	1.710 (1.202–2.368)
Monthly income (ETB)	<1,000	205	9.8	38.2	1	
	1,000–3,000	76	8.6	18.5	0.864 (0.621–1.140)	
	3,001–5,000	35	5.6	9.6	1.053 (0.881–1.340)	
	>5,000	12	2.5	7.2	1.215 (0.991–1.516)	
BMI (kg/m^2^)	Normal (18.5–24.9)	200	13.1	44.0	1	
	Underweight (<18.5)	28	3.0	1.9	1.805 (1.531–2.199)	
	Overweight (25–29.9)	69	6.7	23.4	1.915 (1.691–2.212)	
	Obese (≥30)	31	3.7	4.2	2.205 (1.981–2.514)	
Type of diabetes	T1DM	147	11.7	25.2	1	
	T2DM	181	14.8	48.3	1.603 (1.385–1.919)	
Duration of diabetes (years)	<5	131	9.4	30.5	1	1
	5–10	108	4.6	28.4	0.713 (0.563–0.917)*	0.325 (0.253–0.614
	>10	89	12.5	14.6	0.602 (0.443–0.819)*	2.585 (2.321–5.807)**
Hypertension	Absent	231	14.9	55.5	1	1
	Present	97	11.6	18.0	2.392 (1.429–4.006)*	2.390 (1.394–4.099)**
Smoking habit	Smoker	3	0.0	0.9	0.796 (0.446–1.478)	
	Non-smoker	325	26.5	72.6	1	
Alcohol consumption	Alcohol non-consumer	308	25.3	68.7	1	
	Alcohol consumer	13	0.9	3.0	0.673 (0.473–0.903)	
	Alcohol ex-consumer	7	0.3	1.8	0.724 (0.423–1.072)	
Regular physical activity (days/week)	None	314	26.2	69.5	1	
	1–2	7	0.0	2.1	0.322 (0.024–0.671)	
	3–4	6	0.3	1.5	0.421 (0.275–0.772)	
	>5	1	0.0	0.3	0.520 (0.328–0.870)	
Family history of kidney disease	Absent	293	21.9	67.4	1	1
	Present	35	4.6	6.1	2.302 (1.120–4.731)*	2.884 (1.338–6.218)**
SBP (mm Hg)	Normal (<120)	165	11.6	38.7	1	1
	Elevated (120–129)	50	4.3	10.9	0.671 (0.416–0.966)*	0.693 (0.520–0.904)
	Stage 1 (130–139)	45	4.6	9.1	0.774 (0.507–1.008)*	0.790 (0.622–1.009)
	Stage 2 (≥140)	68	6	14.8	0.875 (0.717–1.068)*	0.894 (0.724–1.104)
DBP (mm Hg)	Normal (60–79)	239	13.3	59.6	1	
	Stage 1 (80–89)	60	9.6	8.6	0.511 (0.416–0.862)	
	Stage 2 (90–109)	29	3.6	5.2	0.601 (0.423–0.912)	
FBG (mg/dl)	<150	146	12.5	32	1	
	≥150	182	14.0	41.5	0.743 (0.520–0.992)	

AOR, adjusted odds ratio; CI, confidence interval; CKD, chronic kidney diseases; COR, crude odds ratio; DBP, diastolic blood pressure; ETB, Ethiopian birr; FBG, fasting blood glucose; SBP, systolic blood pressure; T1DM, type 1 diabetes mellitus; T2DM, type 2 diabetes mellitus.

* *p*-value < 0.25; **p-value < 0.05.

## Discussion

This study showed that 26.5% of patients with diabetes mellitus had CKD. Factors found to be associated with CKD in a multivariable logistic regression model were hypertension, longer duration on diabetes, and having a family history of chronic kidney diseases.

The finding of this investigation is consistent with the prevalence of CKD among diabetic patients in Northeast Ethiopia, which was reported to be 26.3% (15). In addition, the prevalence is in line with studies conducted in Northern Thailand (24.4%) (17), but pooled prevalence was observed in Africa (24.7%) (18) and the Middle East region (28.96%) (19). In comparison to earlier studies carried out in Ethiopia, the current study's result was higher: 16.7% in Bahir Dar (20), 14.3% in Gondar (21), 2.7% in Jinka (22), and 18.2% in Butajira (13). The observed discrepancies could be caused by differences in the eGFR calculation equation and the criteria used to define CKD. In contrary, the result of our study was lower than the prevalence in Southwest Nigeria (39.8%) (23). Differences in study setting, sample size, and ethnicity may have contributed to the observed discrepancies. The study also used a more sensitive method to detect albuminuria, which aided in identifying more cases.

In our study, concomitant hypertension was independently associated with the presence of CKD (*p* = 0.002). Among 97 diabetes patients with hypertension, 39.18% had CKD, and it was 2.390 times more likely to have CKD as compared with diabetic patients without hypertension. This is consistent with other related studies that showed hypertension was an associated factor for CKD in patients with type 2 diabetes mellitus (24, 25). Hypertension can cause the arteries surrounding the kidneys to narrow, weaken, and harden. These damaged arteries are unable to send sufficient blood to the renal tissue. As a result, blood pressure regulation is a vital and critical component in avoiding and slowing the advancement of chronic kidney disease.

Duration of diabetes >10 years was independently associated with the development of CKD (*p* = 0.001). Among 89 patients who have had long duration of diabetes (>10 years), 46% developed CKD. Our study findings were consistent with others in this regard (13–15). Evidence indicated that having diabetes for a long time leads to a buildup of advanced glycation end-products, which play a role in causing diabetic kidney problems and other microvascular disorders (26). In another way, as the disease progress over time, *β*-cell function and insulin secretion decrease. This in turn facilitates for the advancement of CKD among the patients (12).

In addition, the family history of CKD was independently associated with CKD (*p* = 0.007) in which the odds of developing CKD among the study participants were 2.884 times increased as compared to no family history of kidney diseases. Thus, this finding reveals that the family history of kidney disease may be useful for early identification of individuals at high risk of CKD. Our study is in line with the study conducted among diabetic patients in a tertiary hospital of Nepal (25) and Southern Ethiopia (13). As described in a study conducted among Koreans, higher risk of kidney disease with an affected family member indicates that shared environment and shared genes likely contribute to kidney disease (27).

### Limitations of the study

Our study had a number of limitations. First, since it was a facility-based study, it might not reflect the true prevalence of CKD in patients with diabetes in the community. Second, we used a cross-sectional study design. Therefore, we could not establish any causal relationship between CKD and its risk factors. This study, therefore, proposes that a longitudinal study with representative samples from health facilities and communities should be conducted to provide up-to-date data for policymakers and program planners. Furthermore, we considered a descriptive multivariable modeling approach without considering the causal dependencies between the variables in the model. Third, even though albumin creatinine ratio is a stronger indicator of CKD, we assessed proteinuria using semiquantitative methods (dipstick), which may affect the reliability of the results due to its low specificity and sensitivity. Another limitation of our study is that we used average blood glucose over 3 months, and this might not represent the true glycemic status. HbA1c would have been a better option to assess glycemic control. Hence, the results should be interpreted with caution.

## Conclusion

The study revealed that one in four diabetic patients had chronic kidney disease. Routine CKD screening should be implemented in patients with diabetes for early detection and delayed progression of CKD. Special attention should be given to patients with a family history of CKD, long duration on diabetes, and concomitant hypertension.

## Data Availability

The raw data supporting the conclusions of this article will be made available by the authors, without undue reservation.
