# Supplementary material for: Prevalence of chronic kidney disease and associated factors among adult diabetic patients: a hospital-based cross-sectional study
Source: Front Epidemiol. 2024 Nov 19;4:1467911. doi: 10.3389/fepid.2024.1467911 (PMC11611590; doi:10.3389/fepid.2024.1467911)
Supplement: Supplementary file 1 [file Table1.docx]

**Supplementary Material**

**Supplementary figure 1**

Figure 1 Prevalence of chronic kidney disease by stage among diabetic patients at Ayder Comprehensive Specialized Hospital, Northern Ethiopia, 2024
